# Cysteine: A Novel Neural Inducer for Rat Bone Marrow Mesenchymal Stem Cells 

**Published:** 2014-05-25

**Authors:** Malek Soleimani Mehranjani, Milad Falahat Chian

**Keywords:** Mesenchymal Stem Cell, Neural Induction, Cysteine, Nestin, β-Tubulin III

## Abstract

**Objective:**

Mesenchymal stem cells (MSCs) can differentiate into various cell types. Since cysteine has structural similarities to neuronal inducers β-mercaptoethanol and glutathione, we examined its effect on neural induction of rat bone marrow MSCs.

**Materials and Methods:**

In this experimental study, cells were treated in a medium containing 1mM cysteine for 24 hours prior to treatment with neuron inducing medium containing 10 mM cysteine for 1, 2 and 3 hours. Cell viability and morphology were assessed by 3-(4,5-dimethylthiazol-2-Yl)-2,5-diphenyltetrazolium bromide (MTT) assay and, Hoechst, propidium iodide and acridine orange staining respectively. Expression of nestin and *β-Tubulin III* genes, as neural cell-specific markers, was studied reverse transcription polymerase chain reaction (RT-PCR). The data was statistically analyzed using One-Way ANOVA and Tukey’s test and p<0.05 was considered significant.

**Results:**

After 3 hours of treatment, neuron like morphology with a considerable expression of nestin and *β-Tubulin III* genes was apparent. The mean cell viability was not significantly different at 1, 2 and 3 hours following induction, compared with the control cells.

**Conclusion:**

Cysteine can induce neural features in rat bone marrow MSCs without reducing cell viability. Therefore, it can be considered as a safer alternative to toxic neural inducer agents such as β-mercaptoethanol.

## Introduction

Mesenchymal stem cells (MSCs) are located in the bone marrow of adult mammals and possess two fundamental properties: the capacity of extensive replication and the potential of differentiation into different cell lineages (1-3). These multipotent cells have also the ability to differentiate into mesodermal tissues including bone, cartilage, fat, tendon and muscle (4-6). These characteristics have made MSCs an appropriate cellular source for tissue engineering applications (7).

MSCs could be induced to differentiate into neural cells under the appropriate differentiation media (8-10). Since the central nervous system (CNS) has limited capacity for self-repair and the loss of its cells generally results in permanent tissue damage, neural development of MSCs could provide a source to treat specific neurological deficits (11- 13).

Exposure of MSCs to agents such as β-mercaptoethanol in serum-free medium induces neuronal morphological features along with the expression of nestin, neuron-specific enolase (NSE), neuron-specific nuclear protein (NeuN), neuron-specific tubulin-1 (TuJ-1) and the mRNA for NSE and Tau protein (14). Meanwhile it has been reported that β-mercaptoethanol is toxic for cells by reducing cell viability (15).

There have been several studies showing that sulfhydryl groups (SH) in compounds such as β-mercaptoethanol, dimethyl sulfoxide (DMSO) and glutathione (GSH) are essential for neuronal induction of bone MSCs (16-18). Cysteine is considered as the most important sulfur containing non-essential amino acid with the chemical formula HO_2_CCH(NH_2_)CH_2_SH (19). Since cysteine contains a –SH group, it can be considered as a neuronal inducer. On the other hand, due to the ability of thiols to undergo redox reactions, cysteine has antioxidant properties which is typical of glutathione, a non-toxic cysteine containing antioxidant (20). Therefore cysteine may have the advantage of being a non-toxic neural inducer agent compared with other toxic inducers such as β-mercaptoethanol.

Therefore this study was designed to investigate the possible effect of cysteine on neural induction of rat bone marrow MSCs by evaluating cell viability, neuron morphological features and the expression of nestin and *β-tubulin III* genes as two important neuron-specific markers.

## Materials and Methods

### Rat MSCs preparation and culture

In this experimental study, adult male Wistar rats 6-8 weeks old (purchased from Tehran Pasteur Institute of Iran and cared under experimental conditions in compliance with National Institutes of Health guidelines for the humane use of laboratory animals) were anesthetized using ether according to the instructions of Arak University Ethical Committee. Both bilateral femurs and tibias were then removed under sterile conditions and placed in Dulbecco’s modiﬁed eagle medium (DMEM, Gibco BRL). MSCs were ﬂushed out with DMEM using a syringe with a 23-gauge needle, followed by gentle pipetting several times to disaggregate cells. Then, the cells were washed two times with DMEM, centrifuged at 2250 rpm for 15 minutes using centrifuge machine model 5810 (Eppendorf, Germany), cultured at a density of 2.5×10^5^/cm^2^ in DMEM supplemented with 15% fetal bovine serum (FBS, Gibco BRL), 100 U/ mL penicillin G, and 100 mg/mL streptomycin (Gibco BRL). The cultures were maintained at 37˚C in humidiﬁed 95% air and 5% CO_2_. After 3 days, non-adherent cells were removed and fresh complete culture medium was added and replaced every 3-4 days. When the cells became 80-90% conﬂuent over 14 days, they were harvested with 0.25% trypsin and 1 mM EDTA (Gibco BRL) for 3 minutes at 37˚C, replated in six-well disk at a density of 1.5×10^5^ / cm^2^ and again grown to near conﬂuence. To expand a culture, the cells were diluted 1:2 per passage. Invert microscopy (Axiocam MR R3, Carl Zeiss, Germany) was used to observe rat MSCs every 2-3 days.

### Cell authentication

In order to confirm the mesenchymal characteristics of cultured cells at the end of the third passage, rat bone marrow MSCs were cultured in 6-well plates at a density of 1×10^5^ cells per well and grown for 21 days in the osteogenic medium containing 10 nM dexamethasone, 10 mM β-glycerophosphate and 0.05 mM ascorbic acid. Afterwards, using Alizarin Red Solution, 10% (v/v) acetic acid and 10% (v/v) ammonium hydroxide, mineralization level of bone matrix was analyzed (21).

### Neuronal differentiation

1×10^4^ cells were cultured in DMEM +15% FBS in each well of a 96 well plate. After 24 hours of incubation, the culture medium was refreshed and incubation was carried out for 1, 2 and 3 hours.

In the case of neural induced cells (cysteinetreated group), the same number of cells were cultured in DMEM +15% FBS. About 24 hours prior to the treatment, the cells were washed with phosphate-buffered saline and the medium was replaced with the pre-induction medium consisting of DMEM, 15% FBS and 1 mM/L cysteine. Subsequently, the pre-induction medium was removed, and the cells were washed with phosphate-buffered saline (PBS) and transferred to neuronal induction medium composed of serum-free DMEM and 10 mM /L cysteine for 1, 2 and 3 hours.

### Measurement of cell viability

Cell viability in both control and cysteinetreated groups was quantitatively measured by 3-(4, 5-dimethy thiazo-2-yl)-2, 5 diphenyltetrazolium (MTT) assay as described by Wang and Cynader (22). In brief, the culture medium was replaced with 1 mg/ml MTT in the DMEM supplemented with 15% FBS. After 4 hours incubation at 37˚C in a humidified atmosphere containing 5% Co_2_, the medium was then removed and the cells were washed three times with ice cold phosphate-buffered saline. 100 μl DMSO was added to each well of 96 well plates to solubilize the formazan. The absorbance of each well was measured at 505 nm by ELISA reader (SCO diagnostic, Germany). Using a standard curve, the number of viable cells was determined (12).

### Cell morphology following neural induction

The plates in both control and cysteine-treated groups were washed with PBS. Chromatin and cytoplasm staining was performed using Hoechst, propiduim iodide and acridin orange. For this purpose, 10 μl of Hoechst solution (1 μg/ml in PBS) was added to each well containing 5000 cells for 3 minutes in dark condition. The cells were then washed with PBS and observed under the fluorescence microscope (Olympus, IX70) in order to detect the nucleus morphology. 10 μl of propidium iodide solution (1 mg/ml in phosphate buffer) were added to the cells stained with Hoechst and after washing with PBS, the cells were re-assessed using a fluorescence microscope (Olympus, IX70). In order to detect the cytoplasm morphology of the cells, 10 μl of acridine orange solution (1 μg/ml in phosphate buffer) was added to each well for 2 minutes in dark condition. The cells were then washed with PBS and observed under the fluorescence microscope (Olympus, IX70).

### RT-PCR analysis

Total RNA was extracted from both induced and non-induced rat MSCs using RNeasy Mini Kit (QIAGEN) according to the manufacturer’s instructions. The quantity and quality of extracted RNA were determined by spectrophotometry in 260 and 280 nm and denaturing gel electrophoresis respectively. Subsequently, 2 μg of total RNA was converted into cDNA by Moloney-murine leukemia virus (M-MLV) Superscript II reverse transcriptase (Fermentas) and Oligo dT18 primers. for polymerase chain reaction (PCR) the primers of nestin, β- tubulin III and β-actin were used (Table 1).

Amplification was performed using Premix Taq PCR Kit (TaKaRa), with a PCR program mentioned as following: an initial denaturing step of 5 minutes at 94˚C, 35 cycles of 94˚C for 45 seconds, 60˚C for 45 seconds, and 72˚C for 60 seconds followed by a final extension step of 5 minutes at 72˚C. To exclude the possibility of genomic DNA presence, control PCR reactions using total RNA as a template was performed.

**Table 1:** Primer sequences and conditions for RT-PCT


Target genessize (bp	Primers(forward, reverse)	AnnealingTm (˚C)

**Nestin660**	F: 5´ ACGGATC CATGGATGGGTTTGCTGATGAG 3´ R: 5´ CAGAATTCAGCCAGAGGG GCAGTTTC 3´	60
**β- tubulin III236**	F: 5´ TGAGGCCTCCTCTCACAAGT 3´ R: 5´ TGAGGCCTC_CTCTCACAAGT 3´	60
**β-actin353**	F: 5´ GCTCGTCGTCGACAACGGCTC 3´ R: 5´ CAAACATGATCT GGGTCATCTTCTC 3´	60


### Statistical analysis

Data was statistically analyzed using One Way ANOVA and Tukey’s test and the difference of means was considered significant at p<0.05.

## Results

### Cultured rat MSCs morphology

**Fig 1:**
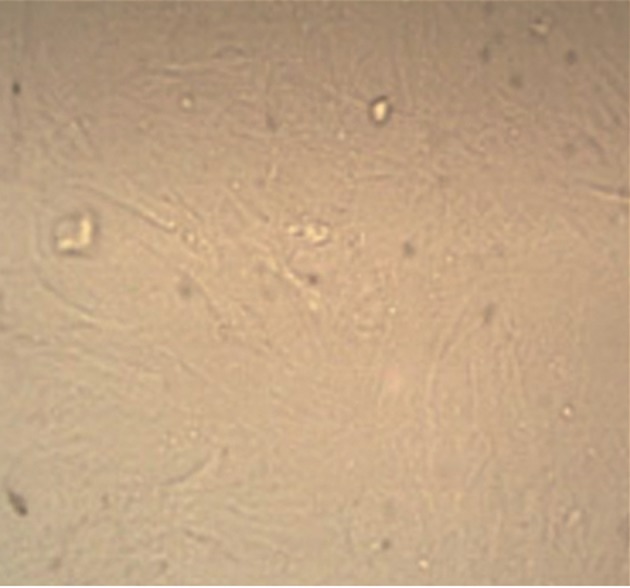
Light micrograph (invert microscope. BEL company) of rat MSCs. Cells exhibited spindle-shaped or large ﬂattened morphology after reaching 90% confluence, original magnification ×20.

The suspended rat MSCs obtained from the bilateral femurs and tibias of adult rats appeared to be small, round and mixed with disclike hematocytes. Within 24 hours the majority of the cells attached to the surface, elongated and spread. Adherent cells exhibited a spindle- shape and ﬂattened morphology following 14 days of culture. Cells grew and exhibited a ﬁbroblast-like morphology when reaching 90% conﬂuence (Fig 1).

### Cell identification

**Fig 2:**
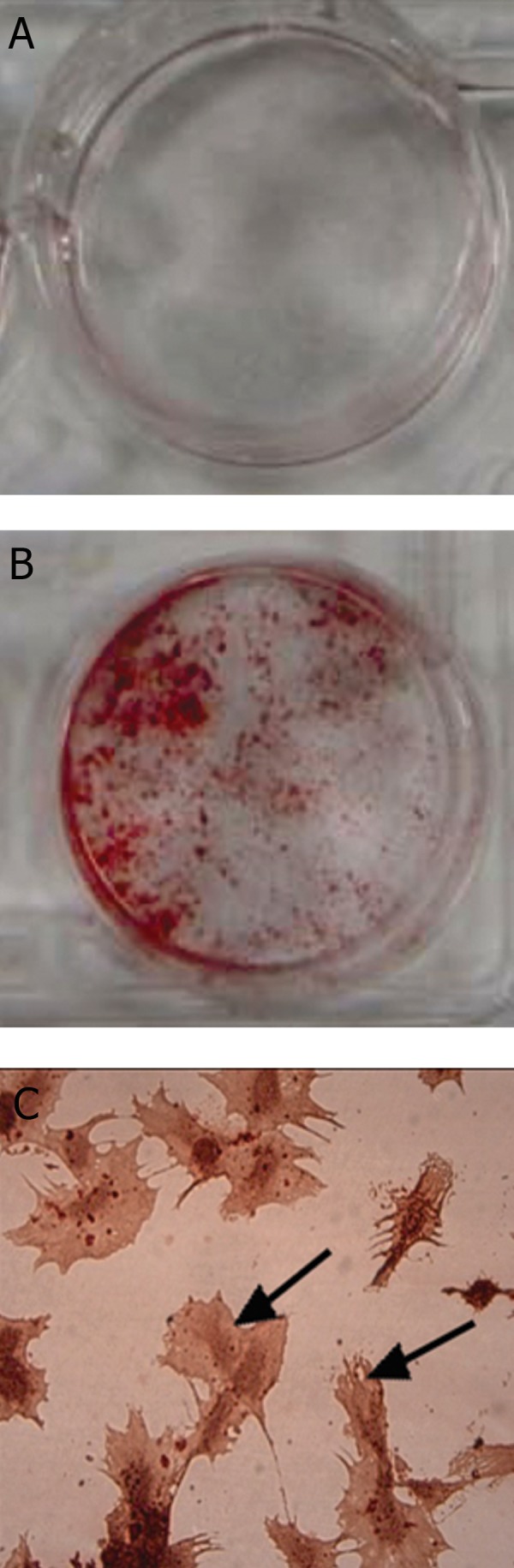
Bone matrix mineralization of rat MSCs cultured in the osteogenic medium. A. Macroscopic image of control cells cultured in non-osteogenic medium (DMEM + 15% FBS). B. Macroscopic image of rat MSCs cultured in the osteogenic medium. C. Micrograph of mineralized matrix of rat MSCs showing mineralized osteoblasts matrix, original magnification ×40.

Alizarin Red staining showed that the rat MSCs cultured in the osteogenic medium were capable of bone matrix mineralization, confirming their mesenchymal characteristics (Fig 2).

### Cell viability

**Fig 3:**
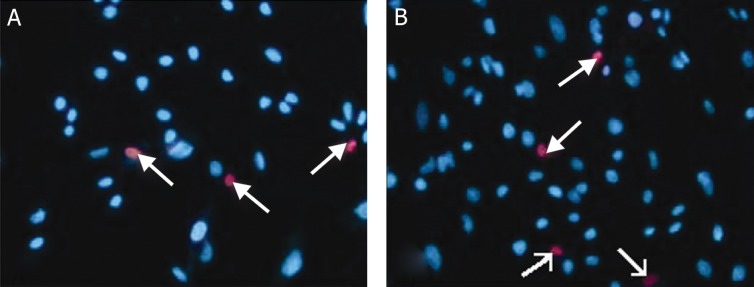
Fluorescent staining (Hoechst and propidium iodide) of rat MSCs cultured in A. Control medium (DMEM + 15% FBS) and B. induction medium (10 mM cysteine + DMEM). The viable and dead cells are shown with blue and red nucleus respectively, original magnification ×40.

The mean number of viable cells in both control and cysteine-treated groups showed no significant difference. Hoechst and propidium iodide staining also revealed that the ratio of the viable to dead cells was relatively the same in both groups (Table 2, Fig 3).

**Table 2:** Comparing the mean number of viable cells in control (DMEM + 15% FBS) and induction medium (10 mM cysteine + DMEM) in different hours. Values are mean ± SD


Medium Time (hour)	Control medium DMEM + 15% FBS (×10^3^)	Induction medium DMEM + 10 mM cysteine (×10^3^)

**1**	8.56 ± 0.43^a^	9.12 ± 0.61^a^
**2**	2 10.5 2 ± 1.34^a^	10.02 ± 0.73^a^
**3**	3 11.5 ± 0.86^a^	11.89 ± 0.83^a^


The means with the same code letters are not considered significantly different from each other (one way ANOVA Tukey’s test, p<0.05).

### The morphology of cysteine-treated cells

The control cells showed spindle-shaped or large ﬂattened morphology while cysteine-treated cells adopted the morphological features typical of neurons such as refractile cell bodies and long branching processes along with the growth of cone-like terminal structures (Figes4, 5).

**Fig 4:**
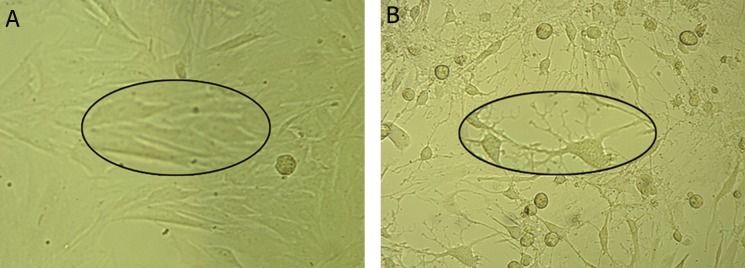
Micrographs of rat MSCs morphology following culture. A. Typical spindle morphology of the cells is observed in the control medium (DMEM + 15% FBS). B. Induction medium (10 mM cysteine + DMEM) caused the cell to show neuron long branching processes, original magnification ×40.

### Analysis of nestin gene expression

The results of RT-PCR revealed that the expression of nestin gene was considerable in the cysteinetreated cells and gave rise to a detectable band, while no gene expression was observed in the control cells. No nestin band formed in the negative control sample (RNA only), confirming the fact that the RNA sample used for RT-PCR contained no detectable genomic DNA (Fig 6).

**Fig 5:**
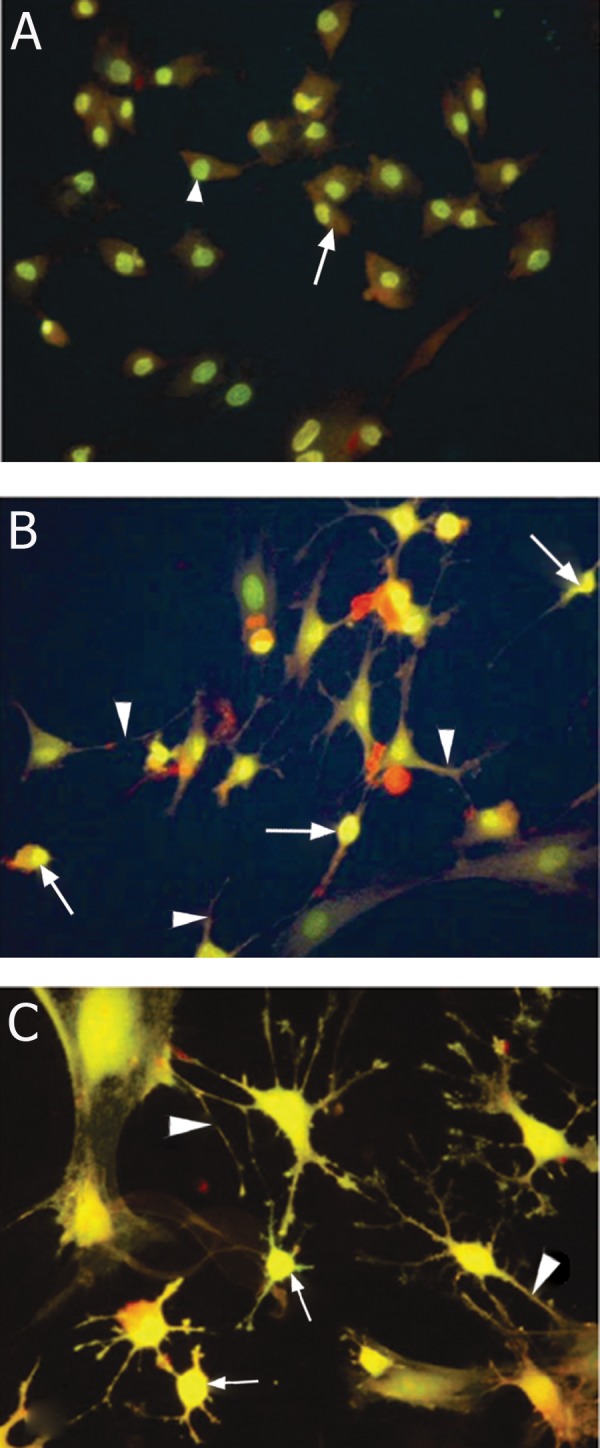
Fluorescent micrographs of rat MSCs stained with acridine orange following culture in A. Control medium (DMEM + 15% FBS) (arrows showing the spindle morphology of cells with a detectable nucleus (arrow head)) and B and C. Induction medium (10 mM cysteine + DMEM) indicating long branching processes (arrow heads) and small cell bodies (arrows), original magnification ×40.

**Fig 6:**
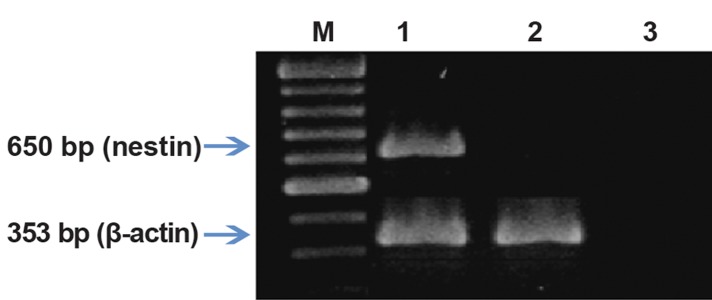
RT-PCR analysis of nestin in rat MSCs treated with cysteine. Lane M; DNA marker, Lane 1; Treated group with cysteine, Lane 2; Control group, Lane 3; Negative control (without cDNA). β-actin was used as housekeeping gene.

### Analysis of β–tubulin III gene expression

**Fig 7:**
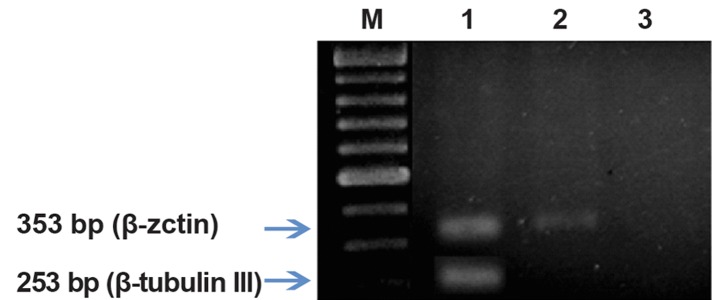
RT-PCR analysis of β - tubulin III in rat MSCs treated with cysteine. Lane M; DNA marker, Lane 1; Treated group, Lane2; Control group, Lane 3; Negative control (without cDNA). β-actin was used as control (housekeeping gene).

β–tubulin III gene expression was positive both before and after treatment with cysteine, but its expression was markedly increased in cysteine-treated cells compared with the control ones. The *β-Tubulin III* band did not form in the negative control sample (RNA only), again confirming the fact that the RNA sample used for RT-PCR contained no detectable genomic DNA (Fig 7).

## Discussion

This study showed for the first time that exposure to cysteine for 3 hours can lead to morphological changes in rat bone marrow MSCs featuring a neuron-like morphology such as refractive cell bodies and long branching processes along with the growth of cone-like terminal structures. Exposure to agents, such as β-mercaptoethanol, DMSO and GSH containing the –SH group can lead to neuronal morphological changes in MSCs. In this case, Woodbury et al. (23) demonstrated the differentiation of human and mice MSCs to neuron-like cells following induction with β-mercaptoethanol, DMSO and butylated hydroxyanisole. Almost 80% of the treated cells exhibited a neuron-like morphology few hours after induction which is due to a breakdown of the actin cytoskeleton and a retraction of the cell edge (24). Since cysteine also contains the sulfhydryl group, it may be deduced that the neuron-like morphology adopted by the exposed rat MSCs has the same mechanism and could be due to the disruption of F-actin and cytoskeletal reorganization. In addition, some studies have argued that MSCs have a dynamic response to induction medium which may not be characteristic of differentiation. The rapidity of which the neuron-like morphology is gained also suggests that the changes in cellular organization and gene expression could not be instigated within the first hours of exposure to inductive conditions (25, 26) and the observed changes in cell morphology could be the result of cytotoxic stress following exposure to inductive agents.

In the present study, the differentiation process of rat bone marrow MSCs was followed by monitoring the mRNA expression of nestin and *β-Tubulin III* genes, two important neuron-specific markers. Expression of nestin and *β-Tubulin III* genes increased in rat bone marrow MSCs following 3 hours of exposure to cysteine, which could be a sign of differentiation in the treated cells.

Other investigations have studied the expression of a wide range of mRNAs and proteins, including those normally reported in terminally differentiated neuronal cells (14, 27, 28). Egusa et al. (29) demonstrated that neuronal cells derived from bone marrow MSCs expressed mRNA species encoding *β-Tubulin III* (an early neuronal marker) and nestin (a neuronal cell marker). In the study carried out by Jiang et al. (28) they reported that rat MSCs can differentiate into neuronal phenotype *in vitro* by expressing cell surface markers speciﬁc to neuron cells such as choline acetyltransferase (ChAT), neurofilaments (NF), glial fibrillary acidic protein (GFAP), Microtubule-associated protein 2 (MAP2), Nestin and *β-Tubulin III* following exposure to β-mercaptoethanol, DMSO and butylated hydroxyanisole.

Similar results were also obtained by Woodbury et al. (15) where they treated rat MSCs with two inductive agents, namely epidermal growth factor (EGF) and basic fibroblast growth factor (bFGF). They showed that the induced cells had a high expression level of NKX6, MAP2, *β-Tubulin III* and nestin genes.

Either way further studies are required in order to understand the underlying mechanism responsible for the observed changes in the cell morphology.

In addition treatment with cysteine did not affect the viability of rat MSCs, while different studies have reported that treatment with some chemical agents containing the thiol group leads to cytotoxic effects and cell death. For instance, Sagara and Makion (8) studied the differentiation and cell viability of bone marrow stromal cells into neurons by administering compounds containing reactive sulfhydryl (SH) group such as 2ME, dithiothreitol (DTT), dithioerythritol (DTE) and GSH. They concluded that GSH did not change the cell viability while 2ME has cell toxic effects thus not a good candidate for clinical applications. The administration of DTT and DTE also caused severe cell death. This could be due to the existence of a pair of readily oxidisable SH groups generating reactive oxygen species under the culture conditions. As a consequence, this may perturb the functions of essential enzymes in the cell, leading to cell death (8).Considering the fact that cysteine is far less toxic compared with other SH containing compounds, it is expected to be more suitable for clinical use in neural induction of MSCs.

## Conclusion

We conclude that cysteine as a member of SH compounds can induce neuron-like morphology with a considerable expression of nestin and *β-Tubulin III* genes in rat MSCs with no significant toxic effects on cell viability. Therefore, we suggest that cysteine is a more suitable candidate for clinical use in the field of regenerative medicine compared with other inductive agents.
